# Resuscitative endovascular balloon occlusion of the aorta deployed by acute care surgeons in patients with morbidly adherent placenta: a feasible solution for two lives in peril

**DOI:** 10.1186/s13017-018-0205-2

**Published:** 2018-09-24

**Authors:** Ramiro Manzano-Nunez, Maria F. Escobar-Vidarte, Claudia P. Orlas, Juan P. Herrera-Escobar, Samuel M. Galvagno, Juan J. Melendez, Natalia Padilla, Justin C. McCarty, Albaro J. Nieto, Carlos A. Ordoñez

**Affiliations:** 1 Clinical Research Center, Fundacion Valle del Lili, Cali, Colombia; 2 Center for Surgery and Public Health - Brigham and Women’s Hospital, Harvard Medical School & Harvard T.H. Chan School of Public Health, Boston, MA USA; 3 Trauma and Acute Care Surgery Division, Department of Surgery, Fundacion Valle del Lili, Cali, Colombia; 4 R. Adams Cowley Shock Trauma Center, Baltimore, MD USA; 5 Trauma Division and Trauma and Emergency Surgery Fellowship, Universidad del Valle, Cali, Colombia; 6 Critical Care Obstetrics, Department of Gynecology and Obstetrics, Fundacion Valle del Lili, Cali, Colombia; 7 School of Medicine, Universidad ICESI, Cali, Colombia

**Keywords:** Placenta accreta, Abnormal placentation, REBOA, Endovascular procedures

## Abstract

Morbidly adherent placenta (MAP), which includes accreta, increta, and percreta, is a condition characterized by the invasion of the uterine wall by placental tissue. The condition is associated with higher odds of massive post-partum hemorrhage. Several interventions have been developed to improve hemorrhage-related outcomes in these patients; however, there is no evidence to prefer any intervention over another. Resuscitative endovascular balloon occlusion of the aorta (REBOA) is an endovascular intervention that may be useful and effective to reduce hemorrhage and transfusions in MAP patients. The objective of this narrative review is to summarize the evidence for REBOA in patients with MAP. We posit that acute care surgeons can perform REBOA for patients with MAP.

## Background

Morbidly adherent placenta (MAP), which includes placenta accreta, increta, and percreta, is characterized by the abnormal invasion of the uterine wall by placental tissue [1]. MAP occurs in 1 per 333 to 533 deliveries and is a leading cause of post-partum hemorrhage (PPH) and maternal mortality worldwide [2, 3]. MAP may manifest at the moment of delivery when the invasive placenta does not readily detach from the uterus, precipitating potentially life-threatening PPH [1, 3].

The optimal management strategy for patients with MAP has been the subject of considerable debate [3–5]. Although several interventions have proven useful and effective for the management of abnormal placentation-associated PPH [1, 3, 6], early identification of patients at risk for catastrophic hemorrhage and aggressive multidisciplinary management is key for improving outcomes [4, 7]. Recent evidence suggests that resuscitative endovascular balloon occlusion of the aorta (REBOA) is a safe and effective intervention to improve hemorrhage-related outcomes in patients with MAP [8–11]. Acute care surgeons have increasing experience in performing REBOA in trauma patients and are optimally suited to, as part of a multidisciplinary team, perform REBOA in women with MAP undergoing either planned or emergent cesarean delivery.

Given the preventable nature of death from PPH, the active participation of acute care surgeons in providing the endovascular (REBOA) care to patients with MAP and at risk of massive hemorrhage could be a cornerstone of effective maternal mortality reduction worldwide, especially in low- to middle-income countries where 99% of all world PPH-related deaths occur [12].

The objective of this narrative review is to summarize the evidence for REBOA in patients with MAP. We posit that acute care surgeons can perform REBOA for women with MAP.

## Morbidly adherent placenta

MAP is the global term encompassing the constellation of conditions resulting from varying levels of abnormal implantation of the placenta: placenta accreta, increta, and percreta. Placenta accreta occurs when the placenta becomes abnormally adherent to the uterine wall. Placenta increta is characterized by further invasion of the abnormal placental tissue into the myometrium. Placenta percreta results when the placenta penetrates the uterine serosa or surrounding tissues [1].

There are well-described risk factors for the development of MAP [1, 3]. The most common and relevant is previous cesarean delivery. Risk for MAP increases with each additional C-section as additional scar tissue develops [13]. The increasing incidence of MAP over the last 60 years has been attributed to increasing C-section rates. Recent estimates are that MAP occurs in 1 in every 300 to 500 pregnancies [2]. MAP thus accounts for a significant proportion of maternal morbidity and mortality cases as it carries a higher risk of catastrophic post-partum hemorrhage, especially in cases of placenta percreta with its deeper penetration [1–3, 14–16].

The primary peril with MAP occurs at the moment of delivery when the abnormally implanted placenta can result in major hemorrhage. Ninety percent of these patients will require transfusion of blood products, and at least 50% will receive a massive transfusion [3, 16, 17]. Despite prenatal surgical planning and perioperative resuscitation, maternal death may still occur in some cases [2, 3, 15].

The current recommendation for the surgical management of MAP at the moment of delivery is to perform a cesarean hysterectomy with the placenta left in situ [3, 5, 18]. Endovascular adjuvant strategies (i.e., prophylactic occlusion balloon catheters in both internal iliac arteries, uterine artery embolization) [19, 20] may be used to prevent and treat bleeding during cesarean hysterectomy. Although these interventions can prove life-saving, a considerable proportion of patients with MAP still end up requiring massive transfusion and post-op obstetrical critical care support. Furthermore, there is no evidence from randomized clinical trials to prefer any surgical intervention over another. Therefore, there is an urgent need for innovation and development of effective strategies to achieve expeditious hemorrhage arrest and prevent the occurrence of massive life-threatening bleeding.

## Resuscitative endovascular balloon occlusion of the aorta in patients with MAP

REBOA is a minimally invasive procedure that introduces a balloon occlusion catheter via a percutaneous groin puncture or cutdown of the femoral artery into the aorta to obtain endovascular aortic occlusion [21, 22]. The physiologic rationale behind the use of REBOA is that it provides proximal control of bleeding and causes volume redistribution from the lower to the proximal vascular beds. These phenomena are hypothesized to ensure an adequate circulating volume and avoid the collapse of systemic circulation.

The use of REBOA in patients with MAP is not new: in 1995, a multidisciplinary team prophylactically deployed an aortic occlusion catheter in a patient with placenta percreta at 34 weeks’ gestation undergoing a planned cesarean delivery. Neither massive transfusion nor admission to the intensive care unit was required, and the patient was discharged 1 week after C-section without any complications [23]. Since that initial case report, additional single cases [24, 25] and uncontrolled case series [10, 26, 27] of MAP patients undergoing C-section combined with temporary aortic balloon occlusion were published. In these reports, REBOA was used either as a rescue maneuver to stop the bleeding during an emergency peripartum hysterectomy [10, 24, 25] or as a prophylactic hemodynamic adjunct in elective cases [26, 27].

Data from retrospective comparative studies suggest that, compared to traditional planned cesarean delivery, planned C-section with the use of REBOA results in better maternal hemorrhage-related outcomes [8, 28–30]. Cui et al. [28] found that intraoperative blood loss was lower in women with placenta previa that underwent planned C-section plus REBOA, compared with patients that underwent C-section alone. These results were consistent with other studies [29, 30] which found that MAP patients benefited from REBOA in terms of average blood loss and transfusions. Moreover, a recent meta-analysis of observational data added additional validation to the finding that the prophylactic use of REBOA during elective C-section results in lower overall blood loss and fewer transfusions [9]. Thus, it seems reasonable to deploy REBOA in emergency and appropriate elective situations in patients with MAP [9].

Despite promising evidence of the potential effectiveness of REBOA in MAP [8, 9], this approach has not been routinely adopted likely due to the required involvement, time, and effort from multidisciplinary teams, including interventional radiologists or trained acute care surgeons, and because of the technical dexterity required.

## REBOA deployed by acute care surgeons in pregnant women with MAP: is this feasible?

Within the field of trauma and emergency surgery, acute care surgeons have typically used REBOA in victims of traumatic truncal hemorrhage who present with hemodynamic instability [31–33]. Trauma surgeons are critical care providers and surgical rescuers [34]; hence, their role in REBOA applicable clinical scenarios has been limited only to those unstable patients who require urgent life-saving surgical interventions. There are, however, other clinical scenarios (i.e., elective care of abnormal placentation) where REBOA has proven feasible, safe, and effective [9], where acute care surgeons could play an active and integral role in the multidisciplinary care of pregnant women affected by MAP [9].

To date, three reports have shown that trauma and acute care surgeons can safely deploy a REBOA in pregnant women with MAP (Table 1) [9, 11, 35]. In these reports, pregnant women with MAP underwent a cesarean delivery with intraoperative REBOA deployment with the goal of either arresting ongoing bleeding [11] or preventing the onset of major hemorrhage [9, 35]. The REBOAs were deployed by acute care surgeons in hybrid suites or conventional operating rooms. No REBOA-related complications were reported, and all patients survived to discharge. Although these reports are case studies without a comparison group, they provide a glimpse into the potential benefits of REBOA in decreasing hemorrhage-related complications by having acute care surgeons be in charge of delivering the endovascular intervention. For example, our group [9] has previously described a case series of 12 pregnant women with a prenatal diagnosis of MAP scheduled for elective cesarean delivery with a multidisciplinary team in which a trauma surgeon was in charge of inserting, deploying, and removing the REBOA (Table 1). In this study, all patients survived, none required either massive transfusion or post-op obstetrical critical care, and no complications relating to REBOA use were noted. This compares with studies of MAP patients which found that they, on average, have blood loss of 3 to 5 l and a significant proportion require massive transfusion [3].

**Table 1:** Studies reporting the deployment of REBOA in MAP patients by acute care surgeons

Author/year	Study type	*N*	Provider	Rem/Prop?	Shock?	Balloon/sheath size	Access technique	Deployment method	Embo after REBOA	Blood loss	Transfusions of PRBCs	REBOA-related complications	Final outcome
Ordoñez 2018	CS	12	Trauma surgeon	Prop	No	Cook CODA/12 Fr	Per guided by US	Fluo	No	1500 ml (900–2750)*	2 U (0–3.5)*	None	All alive
Parra 2018	CR	1	Trauma surgeon	Rem	Yes	NR	NR	Fluo	Yes	Massive bleeding	Activation of the massive transfusion protocol	NR	Alive
Russo 2017	CR	1	Trauma surgeon	Prop	No	ER-REBOA/7 Fr	Per guided by US	External landmarks	No	3000 ml	None	None	Alive

*NR* not reported, *CS* case series, *CR* case report, *Prop* prophylactic, *Rem* remedial, *Per* percutaneous, *Fluo* fluoroscopy, *U* units

*Median (IQR)

Traditionally, MAP patients have received endovascular care from interventional radiologists. Interventional radiologists are trained to deploy endovascular balloon occlusion catheters in the iliac arteries [19] or the aorta [8] and to perform selective arterial embolization when required. Although interventional radiologists’ input in the multidisciplinary care of MAP patients has been remarkable, their participation and skill set demand the availability of modern interventional radiology suites. This infrastructure requirement is available in few well-equipped centers and is scarce in low- and middle-income countries, where PPH continues to be a leading cause of maternal death. In contrast, well-trained and qualified trauma and acute care surgeons can safely deploy a REBOA in a conventional operating room setting [9, 11]. Therefore, acute care surgeons with endovascular training and skills can be consulted either to rescue a pregnant patient who is dying in the operating room due to massive bleeding or to prophylactically deploy and inflate a REBOA in patients undergoing a planned C-section.

With the advent of smaller diameter catheters, REBOA may become more accessible for use in low-resource settings where neither interventional radiology suites nor blood supplies are available. In these scenarios, trauma surgeons with the ability to monitor all stages of REBOA technique may play a crucial role during the multidisciplinary surgical management of patients with MAP by providing the endovascular care which, in turn, could improve morbidity and mortality in these patients.

## The REBOA procedure: how we do it

Previous research has established that the multidisciplinary team management of pregnant women with MAP is associated with a lower likelihood of prolonged maternal admission to the intensive care unit, less large-volume blood transfusion, decreased coagulopathy, fewer ureteral injuries, and less risk for early reoperation [7]. Therefore, in our institution, a multidisciplinary team composed of an obstetrician with training in critical care obstetrics, a urologist, an anesthesiologist, and a trauma and acute care surgeon manages the care of pregnant women with MAP.

With the goal of delivering appropriate care, a defined protocol for planned cesarean hysterectomy with simultaneous REBOA deployment has been established in our institution.

Pregnant women referred to our center with suspicion of MAP are initially evaluated by the obstetrician who makes a diagnosis based on findings from the ultrasonography and the MRI. If prenatal diagnosis of MAP is made, then the patient is scheduled for a planned cesarean delivery. At the time of C-section, the patients are taken to a conventional operating room equipped with a mobile C-arm and an ultrasound machine.

Regarding anesthesia, most centers would place an epidural catheter or use a combined spinal–epidural technique. Although neuraxial anesthesia would allow anesthesia for painless insertion of the REBOA, general anesthesia may be a better option for patients who are predicted to become hemodynamically unstable and require massive resuscitation [36].

After the induction of anesthesia and tracheal intubation, the assisting urologist inserts bilateral ureteral stents with cystoscopic guidance, and the acute care surgeon obtains endovascular access via the common femoral artery for REBOA deployment. Arterial access is obtained via the right groin under ultrasound guidance. The common femoral artery is punctured, and a guidewire is inserted and secured. A sheath (7 Fr) is then passed over the guidewire into the lumen. Finally, the guidewire is withdrawn leaving the introducer sheath in place through which 7 Fr catheter REBOA can be introduced. The depth of REBOA advancement is determined by external landmarks, and C-arm fluoroscopy confirms correct positioning (aortic zone III). With REBOA in place and deflated, the obstetric team starts the cesarean hysterectomy.

The preferred abdominal entry technique is via midline vertical incision, and the hysterotomy is made away from the placenta. After fetal delivery and umbilical cord clamping, the placenta is left in place and the hysterotomy closed using a continuous suture, and a typical cesarean hysterectomy is performed. REBOA can be inflated either at the moment of umbilical cord clamping or when the obstetrician starts dissecting the most vascularized area [9]. We prefer, however, to inflate the REBOA immediately after umbilical cord clamping to provide a less bloody surgical field which in turn may accelerate the hysterectomy. With hysterectomy completed, the REBOA is deflated and removed along with the sheath with manual digital pressure applied to the groin puncture site for hemostasis.

There is currently no standard of care guideline regarding the appropriate care of pregnant women in the REBOA post-catheterization period. However, we recommend close monitoring for at least the first 24 h post-procedure. Examinations should be done every 15 min in the first 2 h post-REBOA and then every hour in the following 6 h. The surgeon in charge should examine the access site and assess extremity color, temperature, and pulses and must be aware of swelling and pain at the vascular access site, as well as of weak or absent distal pulses. Close monitoring during the first hours post-catheterization with the active participation of the bedside nurse is paramount for early identification and management of complications post-REBOA.

## The role of acute care surgeons in MAP surgical protocols: beyond surgical rescue

Acute care surgeons can successfully be integrated into a multidisciplinary team responsible for the elective surgical care of otherwise healthy patients. Traditionally, acute care surgeons have been responsible for patients requiring emergency surgical care. However, with the growth of specific surgical fields, the scope of acute care surgeons has been reduced to simple surgical procedures. For example, Pottenger et al. [37] showed that among the ten procedures that trauma surgeons performed most frequently per year, seven could be performed at the bedside. It is clear that the field of acute care surgery requires expansion of horizons to survive the predominant model of excessive surgical specialization. In that sense, our proposal complements the five pillars of acute care surgery described by Pietzman and colleagues [34] as acute care surgeons can be consulted to rescue pregnant women with ongoing PPH or to provide elective endovascular care to patients at risk of catastrophic PPH.

## Conclusion

REBOA is a feasible and safe surgical intervention that has proven effective in reducing the volume of blood loss and transfusions in pregnant women with morbidly adherent placenta undergoing cesarean delivery. This intervention can be safely delivered by acute care surgeons with endovascular training and experience in a conventional operating room. As PPH secondary to MAP continues to be a leading cause of maternal morbidity and mortality, we believe that our protocol could reduce the burden of mortality attributable to MAP worldwide.

## Abbreviations

MAP: Morbidly adherent placenta
PPH: Post-partum hemorrhage
REBOA: Resuscitative endovascular balloon occlusion of the aorta
